# Supplementary material for: Comparative multiomics analysis of cell physiological state after culture in a basket bioreactor
Source: Sci Rep. 2022 Nov 23;12:20161. doi: 10.1038/s41598-022-24687-4 (PMC9686226; doi:10.1038/s41598-022-24687-4)
Supplement: Supplementary file 1 — Supplementary Information 1. [file 41598_2022_24687_MOESM1_ESM.zip › raw data/Transcriptomic raw data/6.Enrichment/1.GO/TE1vsTC1/UP/TE1vsTC1.up_GOdot.pdf]

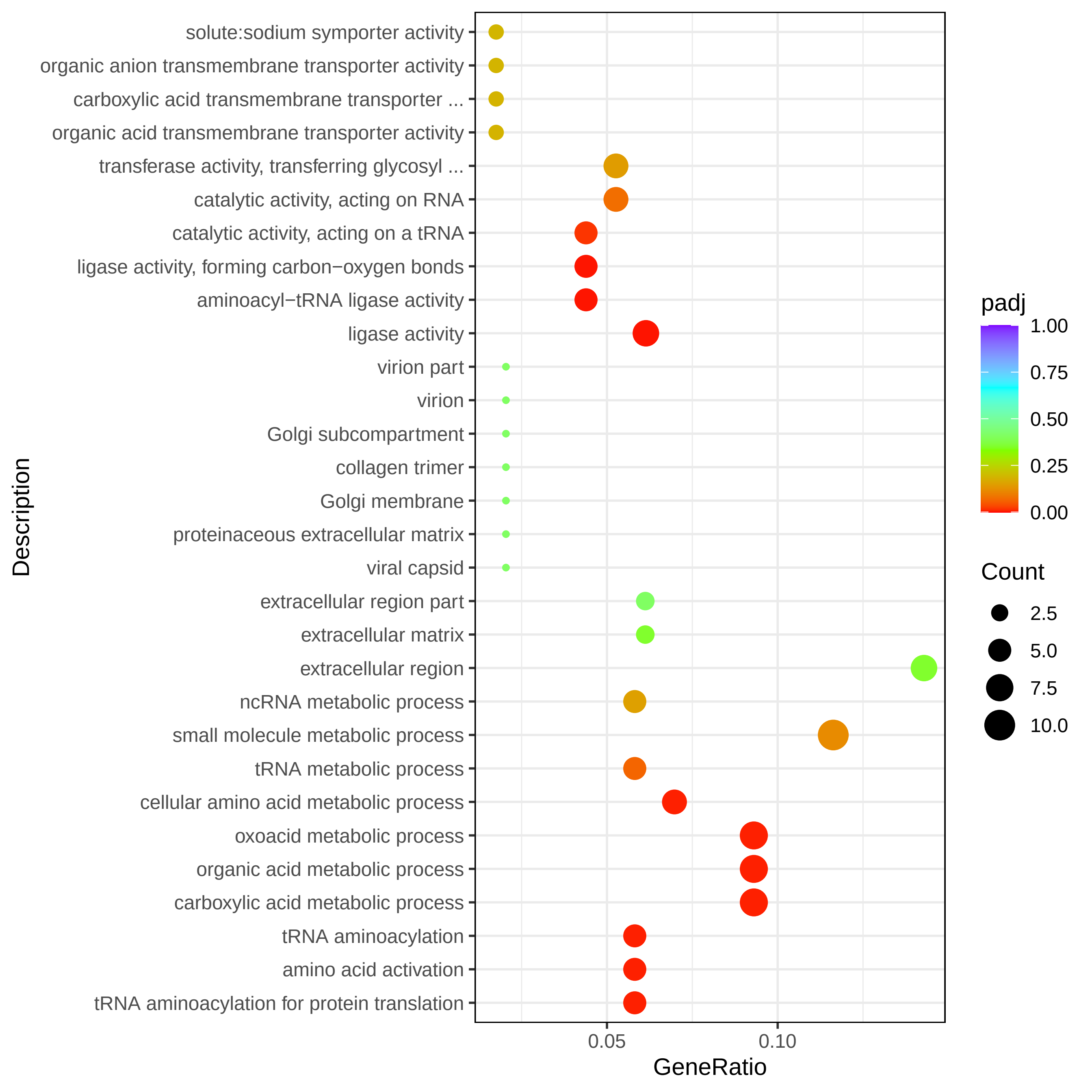

solute:sodium symporter activity
organic anion transmembrane transporter activity
carboxylic acid transmembrane transporter ...
organic acid transmembrane transporter activity
transferase activity, transferring glycosyl ...
catalytic activity, acting on RNA
catalytic activity, acting on a tRNA
ligase activity, forming carbon−oxygen bonds
aminoacyl−tRNA ligase activity
ligase activity
virion part
virion
Golgi subcompartment
collagen trimer
Golgi membrane
proteinaceous extracellular matrix
viral capsid
extracellular region part
extracellular matrix
extracellular region
ncRNA metabolic process
small molecule metabolic process
tRNA metabolic process
cellular amino acid metabolic process
oxoacid metabolic process
organic acid metabolic process
carboxylic acid metabolic process
tRNA aminoacylation
amino acid activation
tRNA aminoacylation for protein translation
Description
0.05
0.10
GeneRatio
padj
1.00
0.75
0.50
0.25
0.00
Count
2.5
5.0
7.5
10.0
